# PrEP Use in Times of COVID-19 in the Netherlands: Men Who Have Sex With Men (MSM) on PrEP Test Less for HIV and Renal Functioning During a COVID-19 Related Lockdown

**DOI:** 10.1007/s10461-022-03693-7

**Published:** 2022-05-16

**Authors:** Lizette C. Krist, Hanne M. L. Zimmermann, Mart van Dijk, Sarah E. Stutterheim, Kai J. Jonas

**Affiliations:** Faculty of Psychology and Neuroscience, Department of Work and Social Psychology, Maastricht University, Universiteitssingel 40, 6229 ER Maastricht, The Netherlands

**Keywords:** COVID-19, Pre-exposure prophylaxis, Men who have sex with men, Netherlands, HIV testing, COVID-19, Profilaxis preexposición, Hombres que tienen sexo con hombres, Países Bajos, Pruebas de VIH

## Abstract

As a result of the COVID-19 pandemic, HIV care and prevention efforts have been disrupted. We investigated pre-exposure prophylaxis (PrEP) use and testing behaviors among MSM in the Netherlands, and the factors that influenced testing behaviors during the COVID-19 pandemic. A cohort of 766 MSM, established in 2017, was asked in August 2020 to report on their experiences during the COVID-19 pandemic via an online survey. Participants (n = 319) reported increased PrEP use and, among PrEP users (n = 211), significantly lower rates of having tested in the last 3 months for HIV and renal functioning compared to before the pandemic. Daily PrEP use and a higher number of sexual partners during the pandemic was significantly associated with continued HIV testing. Continued renal functioning testing was associated with older age. Correcting for pandemic-related disruptions in PrEP use and care will require sustained effort to understand and address missed opportunities.

## Résumé

Como resultado de la pandemia de COVID-19, los cuidados y esfuerzos de prevención de VIH han sido interrumpidos. Investigamos el uso de la profilaxis preexposición (PrEP) y el comportamiento de diagnóstico de HSH en los Países Bajos y los factores de influencia durante la pandemia. Una cohorte de 766 HSH, establecida en 2017, reportó en agosto 2020 sus experiencias mediante una encuesta en línea. Los participantes (n = 319) reportaron un incremento en el uso de PrEP y, entre los usuarios de PrEP (n = 211), tasas menores de pruebas de VIH y funcionamiento renal comparado con tasas pre-pandemia. El uso diario de PrEP y un número mayor de parejas sexuales en pandemia fue asociado con la solicitud continua de pruebas de VIH. La solicitud continua de pruebas de funcionamiento renal fue asociada al incremento de edad. Un esfuerzo constante será necesario para corregir las interrupciones del uso y cuidado asociado a la PrEP.

## Introduction

The COVID-19 pandemic has impacted HIV prevention worldwide [1]. Discussions of how the pandemic would affect prevention efforts, including pre-exposure prophylaxis (PrEP), have been ongoing since the start of the pandemic. Before the start of the COVID-19 pandemic, global trends showed an increase in PrEP use, with promising implications for HIV prevention [1–6]. Theoretically, this means that the COVID-19 pandemic, through measures to ensure physical distancing, could provide an opportunity to reduce HIV infections [3, 5] by reducing opportunities for transmission [7–9]. However, these same measure also affect access to healthcare and thereby prevention methods such as PrEP. Therefore, this article aims to assess the impact of COVID-19 on the PrEP use and engagement with care of men who have sex with men (MSM) in the Netherlands.

While the impact of the pandemic on access to PrEP and PrEP use has been studied, its effects remain unclear. For example, studies from the USA and South Africa report reduced access to PrEP [8–10] while studies conducted in Australia show that access to PrEP remains relatively stable [3, 4]. Simultaneously, studies on PrEP use in Wales, Australia, and the Netherlands, report reductions in PrEP use during the COVID-19 pandemic [3–6, 11, 12]. However, these studies do not report whether this reduction was due to a change in PrEP regimen, for example from daily to on-demand PrEP use [3, 5]. If these reductions are caused by a voluntary change from daily to on-demand PrEP use, it would be possible for people using PrEP on-demand to resume taking PrEP daily as soon as restrictions ease.

According to PrEP guidelines, PrEP care includes not only access to PrEP, but also 3-monthly physician visits for HIV and renal function testing and, depending on the regimen, prescription refills [1, 2, 13–16]. Additionally, PrEP guidelines emphasize the importance of safely stopping PrEP use, which includes counseling and continued HIV and renal function testing [14–16]. This means individuals who change their regimen or stop their PrEP use during lockdowns should continue to have HIV and renal functioning tests. Studies conducted in Australia report that HIV testing continued to be high during the pandemic, especially for PrEP users [4, 7]. Other studies conducted in France, Australia, South Africa, and the USA, report that PrEP users were missing appointments or having difficulty making appointments due to lockdown restrictions [3, 9, 10, 17]. These differences in access to PrEP care during the COVID-19 pandemic show that national PrEP and COVID-19 policies are likely to influence the impact of the pandemic on sexual health. In addition to these policy differences, access to PrEP care is also influenced by socio-demographic and behavioral factors.

Reports on the influence of demographic factors including age, place of residence, and socioeconomic status on sexual health care (uptake) during the pandemic showed contradicting results. For example, a study conducted in Australia found a higher number of casual partners during the pandemic to be associated with younger age [7] while a study conducted in the USA showed older men were more likely to have an increase in their number of anal sex partners [8]. In a study conducted by Hammoud et al. in Australia, age was not found to be associated with PrEP discontinuation [4] but, in an American study, conducted by Sanchez et al. younger people experienced more economic problems and difficulties in being able to access HIV and STI testing during the pandemic [9]. The findings from these studies [4, 7–9] showcase the importance of assessing country-specific demographic variables in context in order to be able to assess, and recover from, the impact of the pandemic.

Behavioral factors such as regular HIV and STI testing and sexual behavior including chemsex (i.e., sexualized substance use), condom use, and sex with (casual) partners have also been assessed in the context of the pandemic [3–7, 9, 11, 13, 18]. Reduced condom use in general [2], and in combination with PrEP use [4], has prompted investigation into condom use during the pandemic. Some reports found no change in condom use [3, 9] while Jongen et al. found a decrease in condom use, specifically during sex with casual partners [5]. The pre-pandemic trends of reduced condom use [2] could lead to an increase in STI and HIV transmission, especially in the context of reduced access to PrEP. Restrictions in movement and travel may have led to reduced opportunities for sex with (casual) partners during the COVID-19 pandemic, but this is likely to vary depending on the local policies.

## The Dutch Context

In the Netherlands, PrEP has been formally available since July 2016. Before that, there was informal PrEP procurement (e.g., via international pharmacies and “buyers’ clubs”). Procurement via general practitioners picked up in January 2018 when generic formulations became available, and since September 2019 PrEP has been available through sexual health centers [2, 19, 20]. At sexual health centers, PrEP is provided at a reduced price and PrEP care is free of charge.

Disruptions of HIV treatment and prevention due to the pandemic have been reported along the prevention cascade [2, 21]. During the first lockdown measures (13th of March to 31st of May 2020), essential healthcare was prioritized. For sexual health centers, this meant a focus on clients with severe STI-related symptoms and those with a strong indication of needing STI testing [22]. PrEP and PrEP care (including renal function, HIV and STI testing) at sexual health centers continued to be largely accessible during the lockdown, but only for those who were already using PrEP [2, 22, 23]. From June onwards, sexual health care was scaled up again, with the majority of sexual health centers reaching 80% of pre-lockdown capacities by September [22, 23]. The trends in sexual health care provided by GPs, while not yet fully documented, are expected to be similar since care provision was affected by COVID-19 related restrictions (e.g., reduced consultations and consultations for priority cases) [2].

The substantial reduction in test services at sexual health centers during the COVID-19 pandemic are expected to have an effect on both the transmission and diagnosis of HIV and other STIs [2, 18, 23]. A study conducted in Rotterdam, the Netherlands, found a reduction in the number of HIV tests completed, lower positivity rates, and an increase in late presentation to clinical care [21]. Similar trends are described for prevention [5, 6, 11]. The majority of longitudinal PrEP studies in the Netherlands facilitate access to PrEP and testing [5, 6]. This means participants of these studies will likely differ from the general population in their experiences with sexual health care during the COVID-19 pandemic [19].

It is possible that access to sexual health care during the COVID-19 pandemic within the Netherlands was unevenly distributed, for example between urban and rural areas [24]. A recent spatial analysis found greater PrEP use in the main urban areas of the Netherlands (Amsterdam, Utrecht, Leiden, the Hague, and Rotterdam) compared to the rest of the country before the pandemic [25]. However, to the best of our knowledge, no analysis of rural–urban differences in access to PrEP care during the COVID-19 pandemic has been conducted in the Netherlands.

## The Present Study

The present study is part of a cohort study among men who have sex with men (MSM) to assess PrEP intentions and experiences before and after the introduction of PrEP in the Netherlands [20, 26, 27]. Participants were recruited through the Dutch PrEP-advocacy website PrEPnu.nl between February 2017 and March 2019. They were invited to three online surveys at 3-month intervals. With the expectation that the COVID-19 pandemic was impacting PrEP use, we contacted the participants of the cohort again in August of 2020 and asked them to complete an additional survey. This data was compared to cohort data collected before the COVID-19 pandemic, allowing us to investigate trends and predictors of PrEP use, as well as determinants for having recently tested for HIV and renal functioning during the pandemic.

The research questions include: (a) How has PrEP use among MSM changed during the COVID-19 pandemic and related lockdowns in the Netherlands?; (b) How have testing rates of HIV and renal functioning changed during the COVID-19 pandemic?; and (c) What factors predict having recently tested for HIV and renal functioning during the COVID-19 pandemic?

This paper reports on the PrEP use and testing behaviors of MSM in the Netherlands, and the factors that influenced their testing behaviors. We expected change in PrEP use, and frequency of testing for HIV and renal functioning, during the COVID-19 pandemic, as well as predictive effects of demographic and behavioral factors such as age and number of sex partners during the pandemic.

## Method

### Participants and Procedure

**Fig. 1:**
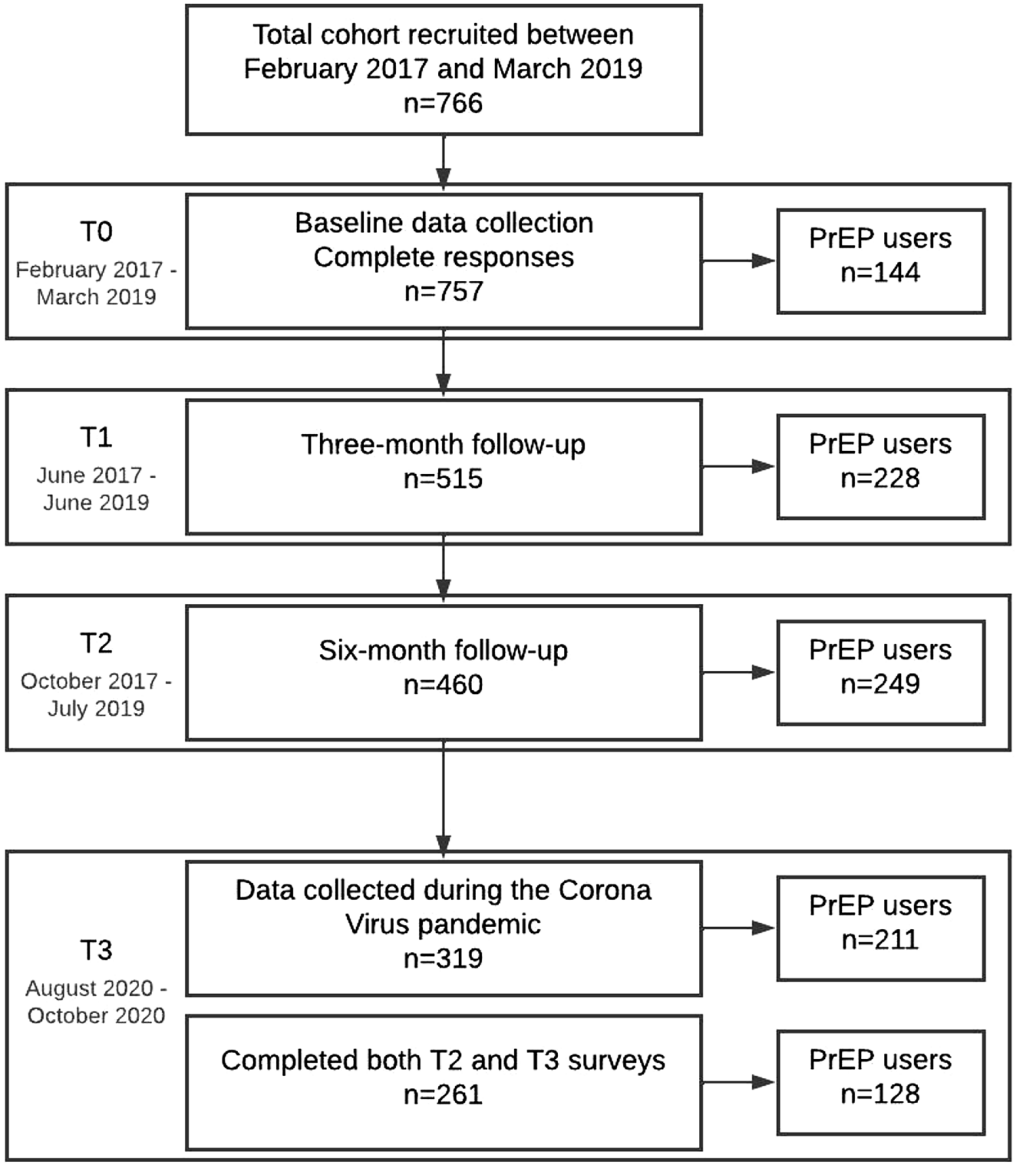
Flowchart of participants per data point

Participants were originally recruited between February 2017 and March 2019 (see Fig. 1). After the initial survey (T0), participants received a follow-up questionnaire at 3 (T1) and 6 months (T2). The full details of the methods of this study are described elsewhere [20].

During the COVID-19 pandemic, in August 2020, participants were contacted again to complete a questionnaire on their sexual behavior and experiences with PrEP during the pandemic (T3). Participants were given the option of entering a raffle to win a €50 gift card.

Participants were asked at all data points about their testing behavior and their use of PrEP. Socio-economic factors such as educational attainment and financial comfort were assessed at T0 only. At T3, participants were asked about their experiences with COVID-19 specifically, and about their place of residence and number of sexual partners in the last 6 months. T3 participants were compared to the entire cohort and found not to be significantly different on any of the measures included below.

This study was approved by the Ethics Review Committee Psychology and Neuroscience of Maastricht University (ERCPN-174_10_ 12_2016).

### Measures

*Age* was assessed with one item; participants were asked at baseline (T0) how old they were.

*Place of residence* was assessed based on the 4-digit postal code, whereby Dutch postal codes of the four largest cities (Amsterdam, Rotterdam, The Hague, or Utrecht) were coded as ‘in core urban area’ and the rest of the Netherlands as ‘outside of core urban area’.

*Financial situation* was assessed at T0 with: “Currently, how would you say you are doing financially?” with answer categories ‘You can’t make ends meet without borrowing’ (1); ‘You are having problems making ends meet’ (2); ‘You are getting by but have to be careful’ (3); ‘Things are alright’ (4); ‘You are doing rather well’ (5); and ‘You are doing really well’ (6). These were dichotomized as ‘not financially comfortable’ (1–3) and ‘financially comfortable’ (4–6).

*Educational attainment* was assessed at T0 with: “Are you attending or have you finished higher education?” which was answered with ‘yes’ or ‘no’.

*Year of first PrEP initiation* was assessed through the following item: “When was the first time you used PrEP?” The earliest listed year of initiation (depending on when participants first responded to the question) was used to determine when participants first initiated PrEP.

*PrEP use* was assessed with: “Are you using PrEP?” to which participants answered ‘Yes, daily’ (1); ‘Yes, intermittently’ (2); ‘Yes, recreationally’ (3); ‘No, but I have used PrEP before (more/less than 6 months ago)’ (4/5); ‘No, I haven’t used PrEP at all’ (6). These categories were dichotomized as ‘yes’ (using PrEP; 1–3) or ‘no’ (not using PrEP; 4–6). Current PrEP users (1–3) were further dichotomized as ‘daily PrEP users’ (1) or ‘not daily PrEP users’ (2–3).

*HIV, STI, and renal function testing* were assessed with three items: “Did you get an HIV/STI/renal test in the last 3 months?”. For all three, answer categories were: ‘Yes’ (1); ‘No and I’m not planning to in the near future’ (2); ‘No, but I’m planning to in the near future’ (3); ‘No, but I tried to’ (4). These were dichotomized as ‘yes’ (1) and ‘no’ (2–4).

*Number of sex partners in the last 6 months* was assessed with the following item: “How many sex partners have you had in the past 6 months?”.

*Sex work* was assessed at T0 with the following item: “Have you ever received money, goods or drugs in exchange for sex?” where ‘Yes, in the past 12 months’ (1); ‘Yes, more than a year ago’ (2); and ‘No, never’ (3) were dichotomized as ‘yes’ (1–2) and ‘no’ (3).

*Perceived frequency of condom use* was assessed with one question: “Would you say that your condom use is high in general?” Answer categories ‘Strongly agree’ (1); ‘Somewhat agree’ (2); ‘Neither agree nor disagree’ (3); ‘Somewhat disagree’ (4); and ‘Strongly disagree’ (5) were dichotomized as ‘high’ (1–2) and ‘low’ (3–5).

*Drug use in a sexual context* was assessed with: “Do you take drugs in a sexual context? (e.g., chemsex parties, smoking/slamming crystal meth etc.)” with answer categories ‘Yes’ and ‘No’.

### Analysis

The change in PrEP use and proportion of HIV and renal function tests before and during the COVID-19 pandemic were assessed with McNemar’s chi-square tests. To assess predictors of having tested in the last 3 months—during the COVID-19 pandemic (T3)—we first conducted univariable logistic regression analyses for HIV and renal function testing separately based on each of the demographic and behavioral factors outlined above. Subsequently, multivariable logistic regressions were performed with those variables that had a *p* value lower than 0.2 in the univariable regression analyses. Backward selection was used to exclude variables from the final model until all included variables had a *p* value lower than 0.05.

## Results

### Participants and Participant Characteristics

All 766 cohort participants were invited to participate at every data collection point. The various sample sizes referred to in the analysis are described in Fig. 1.

PrEP use before the COVID-19 pandemic (T2) was 54.1% (249 of 460); during the pandemic (T3) 66.1% (211 of 319) of participants used PrEP (Fig. 1). During the COVID-19 pandemic (T3), 52.7% (168 of 319) of participants had tested for HIV in the last 3 months, and 68.2% (144 of 211) of PrEP users had tested for HIV (Table 1). For renal function testing 61.1% (129 of 211) of PrEP users reported having tested in the last 3 months at T3; for STI testing this percentage was 68.2% (144 of 211).

**Table 1:** Descriptive statistics of all participants (n = 319) and only those who use PrEP (n = 211) during the COVID-19 pandemic

	T3 participants (*n* = 319)	T3 participants using PrEP (*n* = 211)
Age (years; mean, range)	43 (18–72)	44 (20–71)
Place of residence		
Outside core urban area	146 (45.8%)	97 (46.0%)
In core urban area	135 (42.3%)	95 (45.0%)
Missing^a^	38 (11.9%)	19 (9.0%)
Year of first PrEP initiation		
2010	1 (0.3%)	1 (0.5%)
2013	1 (0.3%)	1 (0.5%)
2014	2 (0.6%)	1 (0.5%)
2015	7 (2.2%)	7 (3.3%)
2016	25 (7.8%)	21 (10.0%)
2017	63 (19.7%)	49 (23.2%)
2018	89 (27.9%)	70 (33.2%)
2019	48 (15.0%)	39 (18.5%)
2020	23 (7.2%)	20 (9.5%)
Missing^a^	60 (18.8%)	2 (0.9%)
PrEP use		
Yes, I use PrEP daily at the moment (every day or via TTSS scheme)	109 (34.2%)	109 (51.7%)
Yes, I use PrEP intermittently (more or less every time I have sex)	66 (20.7%)	66 (31.3%)
Yes, I use PrEP recreationally/on demand (during special phases/moments when I have sex)	36 (11.3%)	36 (17.1%)
No, but I have used PrEP before (less than 6 months ago)	21 (6.6%)	–
No, but I have used PrEP before (more than 6 months ago)	28 (8.8%)	–
No, I haven't used PrEP at all	59 (18.5%)	–
Missing^a^	–	–
HIV testing		
Tested for HIV in the last 3 months	168 (52.7%)	144 (68.2%)
Did not test for HIV in the last 3 months	119 (37.3%)	47 (22.3%)
Missing^a^	32 (10.0%)	20 (9.5%)
Renal function testing		
Tested for renal function in the last 3 months	145 (45.5%)	129 (61.1%)
Did not test for renal function in the last 3 months	142 (44.5%)	62 (29.4%)
Missing^a^	32 (10.0%)	20 (9.5%)
STI testing		
Tested for STI in the last 3 months	171 (53.6%)	144 (68.2%)
Did not test for STI in the last 3 months	116 (36.4%)	47 (22.3%)
Missing^a^	32 (10.0%)	20 (9.5%)
Number of sex partners in the last 6 months (number; mean, SD)	12 (SD = 25.3)	14 (SD = 18.3)
Perceived high frequency of condom use		
Strongly agree	30 (9.4%)	15 (7.1%)
Somewhat agree	40 (12.5%)	24 (11.4%)
Neither agree nor disagree	42 (13.2%)	26 (12.3%)
Somewhat disagree	59 (18.5%)	39 (18.5%)
Strongly disagree	123 (38.6%)	93 (44.1%)
Missing^a^	25 (7.8%)	14 (6.6%)
Drug use in a sexual context		
Yes	139 (43.6%)	107 (50.7%)
No	148 (46.4%)	84 (39.8%)
Missing^a^	32 (10.0%)	20 (9.5%)

Data collected during the COVID-19 Pandemic (T3)

^a^Missing data due to skipped question or illegible response

### Main Results

**Fig. 2:**
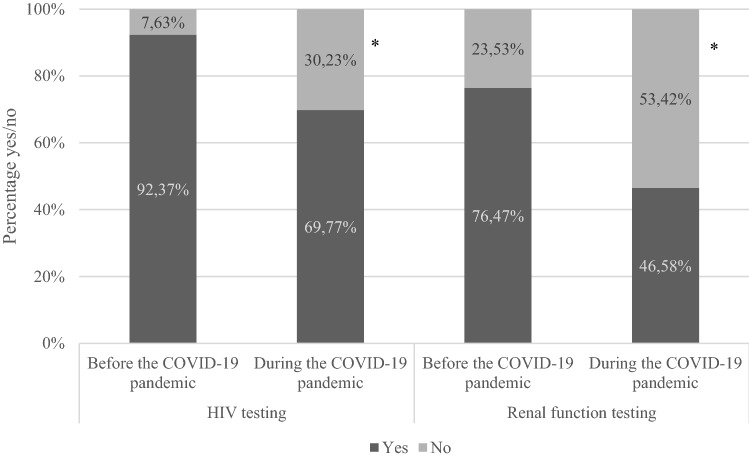
Percentage of participants that tested in the last 3 months. Note. HIV testing and renal function testing percentages have been calculated based on the total number of participants that answered both the T2 and T3 surveys and were using PrEP at both times (n = 128). * McNemar’s tests showed that both changes in proportions were significant (χ2(1) = 8.83, p = .003 and χ2(1) = 9.50, p = .002)

To assess how the PrEP use of MSM in the Netherlands changed during the COVID-19 pandemic, we ran a descriptive analysis first. Table 2 outlines PrEP use and regimen before (T2) and during the COVID-19 pandemic (T3) among participants that completed both surveys (*n* = 261). A chi-square test showed a significant difference in how participants indicated using PrEP before and during the COVID-19 pandemic (*χ*^2^(25) = 166.07, *p* < 0.001). As Table 2 shows, a smaller percentage of participants indicated discontinuing PrEP before (2.3%) compared to during (16.9%) the COVID-19 pandemic. Despite the lockdown regulations in effect during the COVID-19 pandemic, there was an increase in PrEP users who indicate using PrEP daily (33.7% before and 36.8% during the COVID-19 pandemic). To assess how the COVID-19 pandemic affected testing rates of HIV and renal functioning among participants using PrEP, the testing rates of those who indicated using PrEP at both T2 and T3 (*n* = 128) were compared. Figure 2 shows the difference in the proportion of PrEP using participants that indicated having tested in the last 3 months for HIV or renal functioning before and during the COVID-19 pandemic. McNemar tests showed that both HIV testing and renal function testing decreased significantly during the COVID-19 pandemic, compared to before the COVID-19 pandemic (*χ*^2^(1) = 8.83, *p* = 0.003 and *χ*^2^(1) = 9.50, *p* = 0.002, respectively).

**Table 2:** PrEP use before and during the COVID-19 pandemic (n = 261)

	Before the COVID-19 pandemic (T2)	During the COVID-19 pandemic (T3)
Do you use PrEP?		
Yes, I use PrEP daily at the moment^a^	88 (33.7%)	96 (36.8%)
Yes, I use PrEP intermittently^a^	43 (16.5%)	58 (22.2%)
Yes, I use PrEP recreationally/on demand^a^	27 (10.3%)	24 (9.2%)
No, but I have used PrEP before^b^ (less than 6 months ago)	5 (1.9%)	18 (6.9%)
No, but I have used PrEP before^b^ (more than 6 months ago)	1 (0.4%)	26 (9.96%)
No, I haven't used PrEP at all	97 (37.2%)	39 (14.9%)

Only participants that completed both the T2 and the T3 survey were included in this table

^a^Used to calculate current PrEP use

^b^Used to calculate PrEP discontinuation

Table 3 shows the factors that are related to having tested in the last 3 months for HIV and renal functioning during the COVID-19 pandemic according to univariable and multivariable logistic regression analyses. Place of residence, financial comfort, educational attainment, using PrEP for a longer time, having done sex work, reporting high condom use, and reporting drug use in sexual contexts were not associated with having tested in the last 3 months for either HIV or renal functioning.

**Table 3:** Factors associated with having tested in the last 3 months during the COVID-19 pandemic among current PrEP users (n = 211)

Variables	Univariable model		Multivariable model	
	OR [95% CI]	p-value	aOR [95% CI]	p-value
HIV testing				
Age	1.00 [0.97,1.03]	0.746		
Residence: in core urban area	1.02 [0.51,2.03]	0.966		
Financially comfortable^a^	0.97 [0.39,2.44]	0.949		
Attending or finished higher education^a^	0.73 [0.28,1.92]	0.525		
Years since first PrEP initiation	0.81 [0.63,1.06]	0.120		
Daily PrEP use	7.04 [3.16,15.68]	< 0.001***	5.15 [1.68,15.78]	0.004**
Tested for renal functioning in the last 3 months	70.72 [22.79,219.49]	< 0.001***	77.37 [22.63,264.47]	< 0.001***
Number of sex partners in the last 6 months	1.06 [1.02,1.10]	0.005**	1.05 [1.00,1.09]	0.031*
Having done sex work^a^	3.58 [0.45,28.56]	0.228		
Perceived high condom use	0.89 [0.40,2.01]	0.785		
Drug use during sex	1.84 [0.95,3.57]	0.073		
Renal function testing				
Age	1.02 [0.99,1.05]	0.148	1.06 [1.01,1.10]	0.018*
Residence: in core urban area	1.33 [0.70,2.52]	0.387		
Financially comfortable^a^	1.11 [0.48,2.56]	0.801		
Attending or finished higher education^a^	0.59 [0.24,1.45]	0.249		
Years since first PrEP initiation	0.97 [0.77,1.20]	0.751		
Daily PrEP use	3.70 [1.04,7.05]	< 0.001***		
Tested for HIV in the last 3 months	70.72 [22.79,219.49]	< 0.001***	92.92 [27.53,313.59]	< 0.001***
Number of sex partners in the last 6 months	1.00 [0.99,1.02]	0.694		
Having done sex work^a^	2.42 [0.51,11.39]	0.265		
Perceived high condom use	1.44 [0.65,3.20]	0.368		
Drug use during sex	1.74 [0.95,3.21]	0.076		

STI testing was excluded due to its high correlation with HIV testing. Variables with a *p* value lower than 0.2 were used to perform the multivariable logistic regression. Backward selection was used to exclude variables with a *p* value greater than 0.05 to create the final model

HIV: χ^2^ [3, *N* = 190] = 120.82, *p* < 0.001, Nagelkerke *R*^2^ = 0.697; Renal function: χ^2^ [2, *N* = 190] = 107.24, *p* < 0.001), Nagelkerke *R*^2^ = 0.600

*OR* odds ratio; *CI* confidence interval; *aOR* adjusted odds ratio

^a^Data collected at baseline (T0)

*p < 0.05 **p < 0.01 ***p < 0.001

In the multivariable models, having tested for HIV in the last 3 months was associated with having tested for renal functioning in the last 3 months (aOR 77.37, 95% CI [22.63, 264.47], *p* < 0.001). Having tested for HIV in the last 3 months was also associated with a higher number of sex partners in the last 6 months (aOR 1.05, 95% CI [1.00, 1.09], *p* = 0.031) and using PrEP daily (aOR 5.15, 95% CI [1.68, 15.78], *p* = 0.004) rather than intermittently or on demand. Having tested for renal functioning in the last 3 months was associated with having tested for HIV in the last 3 months (aOR 92.92, 95% CI [27.53, 313.59], *p* < 0.001), as well as being older (aOR 1.06, 95% CI [1.01, 1.10], *p* = 0.018).

A similar trend was observed in the logistic regression analysis conducted with only those participants that completed both the T2 and T3 survey and were using PrEP at both time points (See Table 4 in Appendix).

## Discussion and Conclusion

To assess PrEP use and engagement with PrEP care during the COVID-19 pandemic in the Netherlands, we investigated the PrEP use and testing behaviors of a cohort of MSM surveyed in August 2020.

Our findings show greater PrEP use during the COVID-19 pandemic but a significant decrease in HIV and renal function testing among PrEP users. This reduction is particularly remarkable because of the high testing rates of the participants in this cohort before the COVID-19 pandemic (92.0% and 80.9% completed testing for HIV and renal functioning in the past 3 months respectively). Other studies conducted in the Netherlands have found similar reductions in testing for HIV and STIs [6, 11, 21]. Xiridou et al. conducted a modeling study on the effect of decreased testing among MSM in the Netherlands, during the pandemic [18]. This study predicted that a large decrease in testing for STIs could lead to an increase in Chlamydia trachomatis specifically, even with reduced sexual contacts [18]. Renal function testing during the COVID-19 pandemic has not been previously assessed to our knowledge, but as this usually co-occurs with HIV testing for PrEP users in the Netherlands, trends are expected to match [22, 23].

The trend we found for continued PrEP use and relatively low testing rates during the COVID-19 pandemic is in accordance with findings from other studies. For example, Adam et al.’s study on behavior and sexual health of MSM in the Netherlands during the pandemic found that participants re-engaged in sexual activity and PrEP use rapidly after lockdown restrictions ended, but were slower in returning to STI and HIV testing [11]. This trend is surprising because PrEP care was scaled down but accessible for PrEP users throughout the lockdown period in the Netherlands (March to May 2020) [22].

To better understand the predictors of having continued to test for HIV and renal functioning during the pandemic, we assessed sociodemographic and behavioral factors and found that those who did continue testing for HIV or renal functioning during the COVID-19 were likely to have tested for both. This is as expected since HIV, STI, and renal function testing usually occur simultaneously in the Netherlands [23].

For HIV testing specifically, having a higher number of sex partners in the last 6 months and using PrEP daily were also associated with having tested in the last 3 months. Continued PrEP use for people who have more (casual) sex partners during the COVID-19 pandemic was described in other Dutch studies [5, 6]. The role of PrEP regimen in engagement with care is not yet well understood [4, 5, 13]. It is possible that those who are at a higher risk due to having a higher number of sex partners were also using PrEP daily and more likely to continue testing for HIV, which might mean PrEP users are basing their frequency of testing on their perceived risk of getting HIV. Generally, PrEP users change their regimen based on frequency of sexual contacts, something that could become more prevalent during the pandemic or lockdowns [4, 5]. A resulting growing preference for on-demand use would be disconcerting since it could increase suboptimal testing frequencies.

For renal function testing, being older was predictive of having tested in the last 3 months. Guidelines around renal function testing recommend that people with underlying risk factors (such as kidney disease, hypertension, or diabetes) should be monitored carefully [15, 16]. Moreover, being older is a risk factor for developing kidney disease [16]. The findings regarding factors associated with continued testing therefore suggest that participants might be aware of specific risk factors. It is possible that PrEP users or their health care providers are making an assessment of their need and are testing because of a higher perceived risk of testing positive for HIV or renal failure. PrEP users who normally test consistently would be aware that a failure to test for renal functioning despite continued PrEP use could lead to an increase in adverse events and a late presentation of kidney damage [15, 16].

### Strengths and Limitations

This study has several strengths and limitations. To our knowledge, this is the first study to use longitudinal data of participants that are accessing sexual health outside of a clinical trial or study context. It therefore provides insights into the way MSM in the Netherlands have engaged with PrEP during the COVID-19 pandemic.

Due to the nature of the study and the origin of the cohort, we can report data on MSM living outside the main urban area, too. Such data are often lacking in other studies from the Netherlands. The results of this study are not intended to be generalizable to the general MSM population in the Netherlands, but meant to complement the PrEP research cohort data assessed by the Municipal Health Services [5, 22, 23].

In this cohort, not all data were available for all data points, therefore a comparison across all time points was not possible for some indicators. It is also possible that some factors changed between the time of data collection and the COVID-19 pandemic. For example, younger people may have experienced changes in their education level. However, since this affects only a small number of participants (4% of participants were younger than 25), we believe the effect of this to likely be small.

Additionally, the time that passed between the T2 and T3 measure means that changes in PrEP use cannot be ascribed to the COVID-19 pandemic alone. The time of data collection for our pre-pandemic measure (October 2017–July 2019) also denotes that participants were last surveyed on their PrEP use before PrEP became available at sexual health clinics in the Netherlands. It is possible that there was a difference between the participants who completed the T3 survey and those who did not. In order to correct for this possible bias of missing responses, the analyses around PrEP use before and during the pandemic were conducted with only those participants that had completed both surveys. Participants at T3 were compared to the entire cohort and not found to be significantly different.

## Conclusion

Our findings showcase the importance of assessing the impact of the pandemic on sexual health care in context. Encouraging re-engagement with care for those who missed tests for HIV, STIs or renal functioning during the pandemic will require tailored interventions. Future research into the long-term consequences of the COVID-19 pandemic on sexual health should continue to investigate contextual factors that influence uptake and engagement with sexual health care, and how these contextual factors interact with engagement with sexual health care changes throughout time. Additionally, future research should aim to assess the effect of possible interventions in context to mitigate the effects of the pandemic and get back on track with global targets. Our findings and findings from previous studies highlight the importance of maintaining sexual health care during lockdown measures, as well as correcting for missed tests after the easing of lockdown measures. To prevent missed targets of HIV elimination, it is essential to provide adequate PrEP care, even during a pandemic.

## Data Availability

Data and materials can be requested through the corresponding author.

## Appendix

See Table 4.

**Table 4:** Factors associated with having tested in the last 3 months during the COVID-19 pandemic among people using PrEP before and during the pandemic (n = 128)

Variables	Univariable model		Multivariable model	
	aOR [95% CI]	p-value	aOR [95% CI]	p-value
HIV testing				
T1 Age	1.01 [0.97,1.05]	0.716		
T2 Tested for HIV in the last 3 months	1.72 [0.40,7.41]	0.468		
T2 Tested for renal functioning in the last 3 months	0.84 [0.25,2.79]	0.771		
T2 Daily PrEP use	1.99 [0.82,4.83]	0.130		
T2 Condom use	1.35 [0.43,4.24]	0.607		
T2 Drug use	n/a	n/a		
T3 Tested for renal functioning in the last 3 months	128.57 [16.08,1028.27]	< 0.001***	140.58 [16.30,1212.38]	< 0.001***
T3 Daily PrEP use	6.75 [2.32,19.62]	< 0.001***	7.90 [1.90,32.96]	0.005**
T3 Condom use	1.62 [0.43,6.10]	0.476		
T3 Drug use	1.78 [0.74,4.32]	0.199		
Renal function testing				
T1 Age	1.04 [1.00,1.08]	0.060	1.09 [1.02,1.17]	0.013*
T2 Tested for HIV in the last 3 months	1.53 [0.39,6.07]	0.544		
T2 Tested for renal functioning in the last 3 months	2.38 [0.87,6.48]	0.091	6.86 [1.70,27.63]	0.007**
T2 Daily PrEP use	1.51 [0.69,3.30]	0.303		
T2 Condom use	2.07 [0.77,5.56]	0.150		
T2 Drug use	n/a	n/a		
T3 Tested for HIV in the last 3 months	128.57 [16.08,1028.27]	< 0.001***	26.36 [26.33,2574.83]	< 0.001***
T3 Daily PrEP use	2.71 [1.21,6.06]	0.015*		
T3 Condom use	2.08 [0.63,6.81]	0.228		
T3 Drug use	1.99 [0.90,4.37]	0.088		

1. **p* < 0.05

2. ***p* < 0.01

3. ****p* < 0.001

## Code Availability

N/A.
